# Capturing the Interrelationship between Objectively Measured Physical Activity and Sedentary Behaviour in Children in the Context of Diverse Environmental Exposures

**DOI:** 10.3390/ijerph120910995

**Published:** 2015-09-07

**Authors:** Tarun R. Katapally, Nazeem Muhajarine

**Affiliations:** 1 Department of Community Health and Epidemiology, College of Medicine, University of Saskatchewan, Saskatoon S7N 5E5, Saskatchewan, Canada; E-Mail:Nazeem.muhajarine@usask.ca; 2 Johnson Shoyama Graduate School of Public Policy, University of Regina, Regina S4S 7H1, Saskatchewan, Canada; 3 Saskatchewan Population Health and Evaluation Research Unit (SPHERU), University of Saskatchewan and University of Regina, Saskatoon S7N 5E5, Saskatchewan, Canada

**Keywords:** active living research, ecological perspective, urban design, built environment, home environment, social environment, children, moderate to vigorous physical activity, light physical activity, sedentary behaviour

## Abstract

Even though physical activity and sedentary behaviour are two distinct behaviours, their interdependent relationship needs to be studied in the same environment. This study examines the influence of urban design, neighbourhood built and social environment, and household and individual factors on the interdependent relationship between objectively measured physical activity and sedentary behaviour in children in the Canadian city of Saskatoon. Saskatoon’s built environment was assessed by two validated observation tools. Neighbourhood socioeconomic variables were derived from 2006 Statistics Canada Census and 2010 G5 Census projections. A questionnaire was administered to 10–14 year old children to collect individual and household data, followed by accelerometry to collect physical activity and sedentary behaviour data. Multilevel logistic regression models were developed to understand the interrelationship between physical activity and sedentary behaviour in the context of diverse environmental exposures. A complex set of factors including denser built environment, positive peer relationships and consistent parental support influenced the interrelationship between physical activity and sedentary behaviour. In developing interventions to facilitate active living, it is not only imperative to delineate pathways through which diverse environmental exposures influence physical activity and sedentary behaviour, but also to account for the interrelationship between physical activity and sedentary behaviour.

## 1. Introduction

The benefits of physical activity (PA) have been well established, and, independent of PA, sedentary behaviour (SB) has emerged as an important factor that influences a wide range of health outcomes [1,2,3]. Despite this evidence, physical inactivity has reached pandemic levels [4], with the majority of children not accumulating recommended levels of PA [5]. As behavioural interventions directed at individuals have not produced a change at the population level in curbing PA, an ecological perspective called active living research has gained prominence. Active living research is an inter-disciplinary field of study that focuses on the influence of multilevel environmental exposures on PA and SB [6,7]. 

Active living evidence on PA in children has revealed a complex picture, where the roles of multilevel environmental determinants (urban design, neighbourhood built and social environment, school environment, and home environment) on PA have been emphasized [8,9,10,11,12]. Specific to SB among children, initial findings suggest a stronger role of home environment, with parental support and higher socioeconomic status being associated with lower SB [13,14,15,16,17,18,19,20,21,22,23,24]. One finding that has been consistently reported, but not well explored, is low PA among children on weekend days [25,26,27]. Emerging evidence also indicates that independent of PA, SB can be higher on weekend days [27]. Lower PA and SB during weekends could be the result of differential environmental exposure between weekdays and weekend days, and could ultimately influence overall PA and SB (weekdays plus weekend days). 

Although considerable research is being directed to study both PA and SB in children, there is a clear evidence gap regarding the interplay between PA and SB, and how these two behaviours interact with each other within the wider context of varied environmental exposures. A recent meta-analysis by Pearson *et al.* explored the relationship between PA and SB among children and adolescents and concluded that even though SB is inversely associated with PA, these behaviours should not be considered as functional opposites [28]. More importantly, with the increasing usage of accelerometers [27], the entire range of waking activity is being objectively segregated into different intensities of activity (SB, moderate to vigorous PA (MVPA), and light PA (LPA)), where MVPA and LPA together depict total PA. 

Current PA guidelines for children recommend at least 60 minutes of MVPA every day [29]. However, on any given day, children can accumulate this recommended quantity of MVPA, and still remain sedentary for most of the day [30]. To date, SB guidelines focus on only minimizing screen time and not the complete range of SB [31]. Moreover, researchers are now recommending a ‘whole day’ approach to healthy, active living by achieving or exceeding recommended MVPA, minimizing SB and maximizing LPA [32]. 

In developing active living interventions, it is imperative to understand the complex interplay of PA and SB in the context of the environmental exposures that influence them, especially in children and adolescents, as both these behaviours could track into adulthood [13,33]. This study is aimed to examine the influence of urban design, neighbourhood built and social environment, and household and individual factors on the interdependent patterns of objectively measured MVPA, LPA and SB in children aged 10–14 years in the Canadian prairie city of Saskatoon. In exploring these relationships, the influence of weekend activity has been taken into account.

## 2. Materials and Methods

The study is part of an active living research initiative in Saskatoon, Saskatchewan, Canada (www.smartcitieshealthykids.com). The study was conducted in accordance with the Declaration of Helsinki, and the protocol was approved by the University of Saskatchewan’s Research Ethics Board (BEH 14-222).

**Figure 1:**
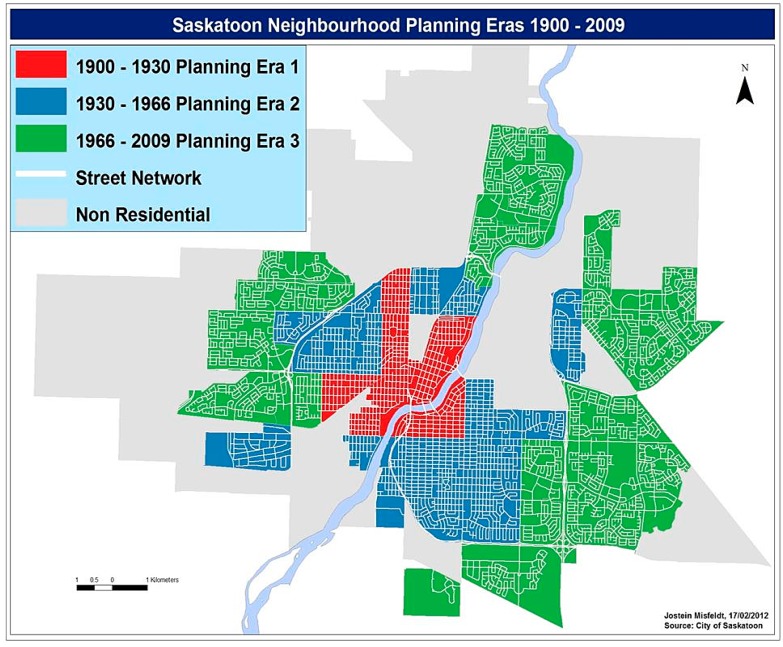
Urban design of Saskatoon depicting the three types of neighbourhoods (grid; fractured grid; curvilinear).

### 2.1. Urban Design of Saskatoon 

Presently Saskatoon’s metropolitan area population of 260,600 is spread across 65 well-defined neighbourhoods [34], where the city plays a major role in urban planning including the geographic allocation of commercial, residential and institutional establishments. In 2010, when urban design data for this study were collected, Saskatoon consisted of 60 residential neighbourhoods. The neighbourhoods designed prior to 1930 surround the city centre and follow a traditional grid-patterned street design (Figure 1—Planning Era 1), typified by higher density, mixed-use neighbourhoods connected by straight, intersecting streets and back alleys. The semi-suburban neighbourhoods built between 1931 and 1966 follow a fractured grid-pattern (Figure 1—Planning Era 2). They are predominantly residential, with lower density and become progressively car-oriented as the distance from the urban centre increases. Finally, the suburban neighbourhoods built after 1967 follow curvilinear street patterns (Figure 1—Planning Era 3), characterized by low-density, almost exclusively residential and highly car-oriented configurations. Working with the City of Saskatoon’s Neighbourhood Planning Department, our Smart Cities, Healthy Kids research team has validated the three types of neighbourhoods belonging to the three different planning eras [35].

### 2.2. Neighbourhood Selection and Recruitment

The neighbourhood selection and recruitment were part of the Smart Cities Healthy Kids initiative. The sampling frame for recruiting children consisted of all 60 residential neighbourhoods in 2010 in Saskatoon categorized into the three types of neighbourhoods (Figure 1). The recruitment was conducted through 30 elementary schools representing all three types of neighbourhoods. The total study sample was representative of all 60 neighbourhoods. Working with our public and Catholic school board partners, we identified four classrooms at each elementary school (grades 5 to 8) for recruitment. After preparing the schools, a letter explaining the study was sent out to children’s primary caregivers through schools with an invitation to participate in the study. Of the 1610 children aged 10–14 years that agreed to participate in the Smart Cities Healthy Kids initiative, 455 children agreed to participate in accelerometry. This study exclusively focuses on children who participated in accelerometry.

### 2.3. Built Environment Measures

In 2009, two validated tools called the neighbourhood active living potential and the Irvine-Minnesota inventory were used to measure specific aspects of built environment [36,37] of Saskatoon. Neighbourhood active living potential is an 18-item tool that was replicated by our team by adding a new dimension called universal accessibility (which measures disabled individuals’ access to built environment) to existing dimensions of safety, density of destinations and activity friendliness [38]. In implementing this tool, pairs of observers independently rated neighbourhood built environment by travelling a predetermined walking route created by random selection and connection of street segments. The inter-observer reliability for neighbourhood active living potential was above 80% [38]. 

Similarly, two observers were employed to administer the Irvine Minnesota Inventory (inter-observer reliability above 70%) to measure the built environment of neighbourhoods in five dimensions: diversity of destinations, pedestrian access, attractiveness, and safety from traffic and crime [37]. In both built environment tools, safety is measured as the observers’ perceived neighbourhood safety.

### 2.4. Census-based Measures

Neighbourhood level socioeconomic variables were derived from 2006 Statistics Canada Census data and 2010 G5 Census projections to account for neighbourhood social environment [39,40].

### 2.5. Individual and Household Data

In 2010, after obtaining written informed consent from parents/guardians on behalf of their children and prior to deploying accelerometers, Smart Cities Healthy Kids questionnaire (Supplementary file) was administered to children to capture their perception of a range of factors (household, parental, peer and neighbourhood) that influence PA. The questionnaire was pilot tested and revised as appropriate prior to field implementation. The questionnaire contained items such as: “In the last 30 days, how often have your family members provided transportation to a place where you can do PA?” and “During a typical week, how often did your friends ask you to walk or bike to school or to a friend’s place?”

### 2.6. Accelerometry

Actical accelerometers (Mini Mitter Co., Inc., Bend, OR, USA) were deployed through schools from April to June in 2010 to capture activity data of 455 children residing in Saskatoon. Children were visited at their respective schools and were asked to wear the accelerometer equipped belt around their waist to maintain proper positioning (*i.e.*, posterior to the right iliac crest of the hip) for 7 consecutive days. They were advised to remove the accelerometers during nighttime sleep and during any water-based activities. The devices were operationalized to measure data at 12:00 a.m. on the day following device deployment (*i.e.*, almost a full day after the device was deployed) to minimize the potential for subject reactivity within the first day of wearing the accelerometer. Accelerometers were pre-programmed to measure movement in 15-second epochs in order to capture the sporadic nature of children’s activity.

The raw accelerometer data were analyzed using KineSoft version 3.3.63 (KineSoft, Loughborough, UK) to derive activity intensities using cut-points specific to the study sample’s age group—SB: <100 counts/minute; LPA: 100 to <1500 counts/minute; MVPA: ≥1500 counts/minute [41,42,43]. The accelerometers and cut-points used in this study are the same as those used in the 2007–2009 Canadian Health Measures Survey [30], whose accelerometry results depicted activity patterns in a nationally representative sample of children in Canada. Furthermore, using the accelerometer sample of the 2007–2009 Canadian Health Measures Survey, operational definitions and data reduction techniques were developed by Colley *et al.* [44]. Valid data for our study were derived by utilizing these population and device-specific (*i.e.*, Actical accelerometers) operational definitions and data reduction techniques, and taking into account established evidence in conducting accelerometry on large samples of children [44,45]. 

Generation of valid data is essential to exclude days of accelerometry from the analysis when the participants do not wear the device for a period of time deemed sufficient to interpret levels of activity [44]. A valid day was defined as a day of accelerometry with 10 or more hours of wear-time [45]. Daily wear-time was estimated by subtracting non-wear-time of a particular accelerometry day from 24 hours. It was determined that non-wear-time would be a period of at least 60 consecutive minutes of zero accelerometer counts, including up to 2 minutes of counts between 0 and 100 [44]. The final sample consisted of children with at least four valid days including at least one valid weekend day, * i.e.*, the valid sample (N: 331; boys: 166; girls: 165 (Age 10: boys: 42; girls: 28) (Age 11: boys: 41; girls: 50) (Age 12: boys: 40; girls: 45) (Age 13: boys: 29; girls: 35) (Age 14: boys: 13; girls: 8)). 

However, even within valid data, there is a chance for systematic variation in daily wear-time, both within (on different days of accelerometer use) and between participants. The systemic variation occurs because even though participants are asked to wear accelerometers from the time they wake up in the morning until the time they go to bed at night, every participant wears or removes the accelerometer at her/his discretion, thus potentially introducing a random or non-random measurement bias to activity measurement. We have previously developed a methodology to control for wear-time variation and minimize measurement bias by standardization of valid data [27]. The same methodology has been replicated in this study to standardize valid data. We have utilized both unstandardized (not controlled for wear-time) and standardized (controlled for wear-time) valid accelerometer data.

### 2.7. Study Variables

MVPA and SB were the main outcome variables. In terms of predictors, LPA and weekend activity intensities were included as independent variables to understand how LPA influenced MVPA and SB, and to observe if weekend activity significantly influenced MVPA and SB throughout the week. Apart from activity intensities, using data from all the measures mentioned in the above sections, extensive sets of variables were derived for this study. Taking into account the hierarchical nature of data distribution, these variables were segregated into two levels: neighbourhood level variables (Level 2) and individual level variables (Level 1) (Table 1).

### 2.8. Statistical Analyses

First, the valid unstandardized accelerometer data were descriptively analyzed to depict group differences in MVPA, LPA and SB accumulation between children residing in different types of neighbourhoods. Next, differences in MVPA and SB accumulation between weekdays and weekend days were descriptively analyzed. Finally, using only valid standardized accelerometer data that were controlled for wear-time, two fixed effects multilevel logistic regression models were fitted using Hierarchical Linear and Non-linear Modeling software by Bernoulli distribution of the outcome variables—mean MVPA dichotomized at 60 minutes/day (≥60 = high; <60 = low) and mean SB dichotomized at 480 minutes/day (≥480 = high; <480 = low). MVPA was categorized based on current PA guidelines for children that recommend at least 60 minutes of MVPA every day [37]. SB categorization was based on sensitivity analyses that utilized a series of mean SB/day cut-points to determine the level of SB that was protective of overweight or obese weight status (Table S1). 

**Table 1:** Hierarchical classification of derived predictors.

Hierarchy	Type	Examples of Derived Variables	Source
Neighbourhood Level Variables	Urban Design	Grid-Pattern	Urban Planning
		Fractured Grid Pattern	
		Curvilinear	
	Built Environment	Diversity of Destinations	Observation Tools: Neighbourhood Active Living Potential and Irvine Minnesota Inventory
		Density of Destinations	
		Safety from Traffic	
		Safety from Crime	
		Attractiveness	
		Pedestrian Access	
		Universal Accessibility	
		Activity Friendliness	
	Neighbourhood Social Environment	Dwelling Value	2006 Statistics Canada Census and G5 2010 Census Projections
		Dwellings per Acre	
		Household Income	
		Socioeconomic Deprivation Index	
Individual Level Variables	Children’s Perception of Household, Neighbourhood, Peer and Parental factors	Transportation Support from Family	Smart Cities Healthy Kids Questionnaire
		Peer Support to Walk or Bike	
		Household Socioeconomic Status	
		Parents’ Education	
	Activity Measures	Moderate to Vigorous Physical Activity	Accelerometry
		Light Physical Activity	
		Sedentary Behaviour	

Before fitting these models, utilizing neighbourhood and individual level variables (Table 1), separate bivariate analyses were conducted to identify significant predictors at both levels. Only bivariately significant predictors were used in the multilevel logistic regression models. In multilevel regression models, model 1 depicts the influence of neighbourhood level variables and model 2 is the final model depicting the influence of both neighbourhood and individual level variables. Only significant results from the final model are presented here. 

## 3. Results

**Table 2:** Descriptive characteristics of the valid study sample depicted across urban design.

Variables	Total	Grid	Fractured Grid	Curvilinear
Sampled Schools	30	6	10	14
Total Sample	331	95	100	136
Boys	166	45	53	68
Girls	165	50	47	68
Age 10	70	16	25	29
Age 11	91	32	22	37
Age 12	85	27	26	32
Age 13	64	13	23	28
Age 14	21	7	4	10
Mean Age (SD; Min,Max)	11.6 (1.1; 10,14)	11.6 (1.1; 10,14)	11.5 (1.2; 10,14)	11.63 (1.2; 10,14)
Mean Body Mass Index (SD; Min,Max)	19.9 (4; 13.4,35.9)	19.8 (4.2; 14,35.9)	20.3 (4.2; 13.4,34.3)	19.7 (3.7; 14.2,33.8)
Mean Accelerometer Wear-time/Day (SD; Min,Max)	796.3 (51.1; 653.3,930.2)	794 (53.1; 680.8,930.2)	797 (53.3; 653.3,915)	797.3 (48.1; 684.5,910.6)
Mean MVPA/Day (SD; Min,Max)	71.2 (31.8; 8,234.5)	72.8 (33.7; 8,178.1)	67.3 (32.9; 13.3,234.5)	73.1 (29.4; 16.6,182)
Mean SB/Day (SD; Min,Max)	540.2 (64.8; 317.4,691.3)	537.8 (68.9; 317.4, 682.6)	546 (70.5; 344, 691.3)	537.3 (57; 379.7,663.4)
Mean LPA/Day (SD; Min,Max)	184.7 (38.9; 92.5,311.6)	183.3 (39.1; 104.4,282.5)	183 (40.9; 92.5,311.6)	187 (37.4; 98,294.6)

SD: standard deviation; Min: minimum; Max: maximum; MVPA: moderate to vigorous physical activity; SB: sedentary behaviour; LPA: light physical activity; Accelerometer Wear-time, MVPA, SB and LPA values are expressed in minutes.

The activity intensities (*i.e*., MVPA, LPA and SB) when distributed by urban design (Table 2) showed that children residing in all types of neighbourhoods irrespective of accumulating the recommended levels of MVPA on average (>60 min/day), were sedentary for most part of the day (~9 hours/day). Although there were no significant differences in activity intensities between the different types of neighbourhoods, all children were consistently sedentary. 

When MVPA and SB were segregated between weekdays and weekend days, boys across all age groups consistently accumulated more MVPA than girls on weekdays, whereas this pattern was not repeated on weekend days. Moreover, both boys and girls of all age groups accumulated more MVPA on weekdays than weekend days (Figure 3). However, the findings for 14-year-old category should be interpreted with caution because of the small sample of participants in this age group. In terms of SB, boys and girls of all age groups accumulated more than 500 minutes of SB on both weekdays and weekend days (Figure 3). 

After descriptive analyses, two separate multilevel models were fitted with MVPA and SB as the outcome variables. In each model, the interdependent relationship between the three intensities of activity was explored in the context of urban design, neighbourhood built and social environment, and household and individual factors. 

**Figure 2:**
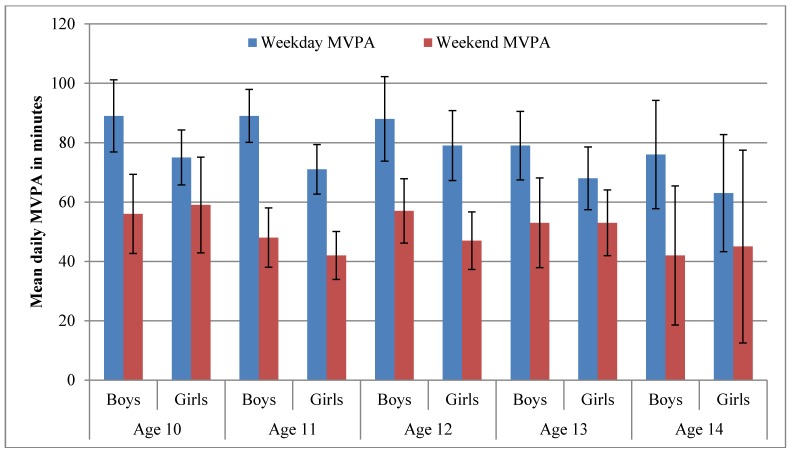
Mean daily moderate to vigorous physical activity on weekdays and weekend days.

**Figure 3:**
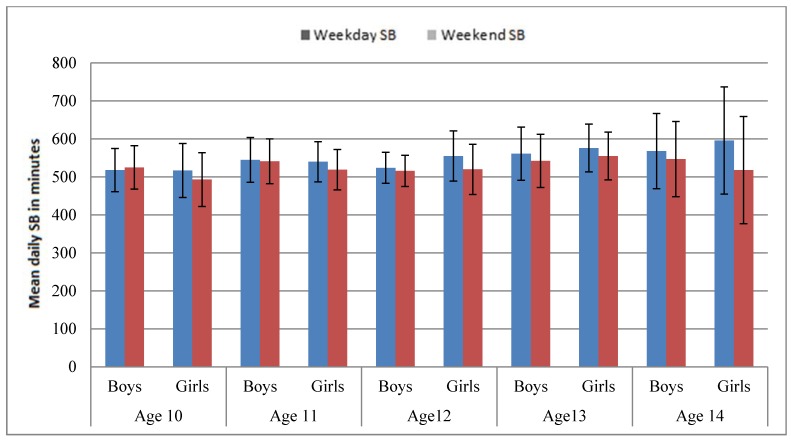
Mean daily sedentary behaviour on weekdays and weekend days.

The MVPA model was fitted to identify factors associated with MVPA accumulation (Table 3). The following children were more likely to accumulate higher MVPA (≥60 minutes/day): Boys (OR = 2.04; CI = 1.15–3.61); children who received frequent (>3 times/week) family transportation (Section 2.5) to a place with physical activity access (OR = 2.34; CI = 1.18–4.67); children who frequently (>3 times/week) walked or biked (Section 2.5) with their peers (OR = 1.90; CI = 1.10–3.29); children who accumulated higher MVPA during the weekend days (OR = 17.24; CI = 6.20–47.97); and, finally, children who accumulated higher LPA (OR = 2.08; CI = 1.15–3.61). 

**Table 3:** Multilevel logistic regression model predicting MVPA accumulation (mean daily MVPA ≥60 *vs.* <60 minutes).

Variables	Null Model	Model 1				Model 2
	OR	CI	OR	CI	OR	CI
Intercept	1.39	1.17–1.72	2.55	1.76–3.80	0.11	0.00–33.48
Fractured Grid *vs*. Grid			0.67	0.42–0.90	0.42 **	0.24–0.89
Curvilinear *vs*. Grid			0.74	0.50–1.11	0.73	0.49–1.68
Diversity of destinations—High *vs*. Low			0.44	0.36–0.78	0.51	0.42–1.30
Boys *vs*. Girls					2.31 **	1.41–3.22
Frequent Family Transport *vs*. Infrequent Family Transport					2.02 **	1.25–3.40
Frequent Active Transport with Peers *vs*. Infrequent Active Transport with Peers					2.13 **	1.60–3.17
High SB *vs*. Low SB					0.18 *	0.10–0.36
High MVPA *vs*. Low MVPA on Weekend Days					19.62 *	10.57–45.47
LPA					2.33 **	1.55–3.20
Age 11 *vs*. Age 10					0.49	0.31–4.92
Age 12 *vs*. Age 10					0.71	0.51–8.36
Age 13 *vs*. Age 10					0.24 *	0.11–0.69
Age 14 *vs*. Age 10					0.25 *	0.16–0.80

OR: odds ratio; CI: confidence interval; MVPA: moderate to vigorous physical activity; SB: sedentary behaviour; LPA: light physical activity; * *p* < 0.05; ** *p* < 0.01; *** *p* < 0.001; frequent family transport and frequent active transport with peers: ≥3 times/week; infrequent family transport and infrequent active transport with peers: <3 times/week; high SB vs low SB: ≥480 minutes *vs.* <480 minutes/day; high MVPA *vs.* low MVPA: ≥60 minutes *vs.* <60 minutes/day; excluding LPA all other variables are categorical.

The following children were less likely to accumulate higher MVPA: children residing in fractured grid-pattern neighbourhoods (Figure 1—Planning Era 2) in comparison with children residing in grid-pattern neighbourhoods (Planning Era 1; OR = 0.40; CI = 0.16–0.97); children who accumulated higher SB ((≥480 minutes/day) (OR = 0.15; CI = 0.06–0.37)) and children aged 13 (OR = 0.44; CI = 0.21–0.90) and 14 (OR = 0.27; CI = 0.08–0.90) years in comparison with children aged 10 years. 

The SB model was fitted to identify factors associated with SB accumulation (Table 4). The following children were less likely to accumulate higher SB (≥480 minutes/day): Aboriginal children (OR = 0.32; CI = 0.14–0.75); children who accumulated higher MVPA on weekend days (OR = 0.18; CI = 0.07–0.46), and children who accumulated higher LPA (OR = 0.50; CI = 0.27–0.95). The following children were more likely to accumulate higher SB: children who accumulated higher SB on weekend days (OR = 10.84; CI = 4.14–28.35), children who wore the accelerometers for longer number of hours each day (OR = 3.98; CI = 2.46–6.44); and, finally, children aged 11 (OR = 2.59; CI = 1.08–6.20), 12 (OR = 3.64; CI = 1.34–9.85), and 13 (OR = 9.26; CI = 2.29–37.32) years in comparison with children aged 10 years. 

**Table 4:** Multilevel logistic regression model predicting SB accumulation (mean daily SB ≥480 *vs.* <480 minutes).

Variables	Null Model		Model 1		Model 2	
	OR	CI	OR	CI	OR	CI
Intercept	3.23	2.75–3.83	3.14	2.28.3.41	0.00	0.00–0.00
Fractured Grid *vs*. Grid			0.81	0.49–1.39	0.97	0.37–2.14
Curvilinear *vs*. Grid			1.03	0.80–2.05	1.20	0.52–2.87
Boys *vs*. Girls					0.74	0.38–1.10
Aboriginal *vs*. Non-Aboriginal Status					0.36 **	0.22–0.73
High SB *vs*. Low SB on Weekend Days					14.92 *	6.73–23.09
High MVPA *vs*. Low MVPA on Weekend Days					0.25 *	0.10–0.44
LPA					0.58 *	0.28–0.88
Age 11 *vs*. Age 10					2.71 **	1.26–5.94
Age 12 *vs*. Age 10					3.52 **	1.84–7.03
Age 13 *vs*. Age 10					9.40 *	2.89–32.59
Age 14 *vs*. Age 10					4.75	0.73–21.62

OR: odds ratio; CI: confidence interval; SB: sedentary behaviour; MVPA: moderate o vigorous physical activity; LPA: light physical activity; * *p* < 0.05; ** *p* < 0.01; *** *p* < 0.001; high SB *vs.* low SB: ≥480 minutes *vs.* <480 minutes/day; high MVPA *vs.* low MVPA: ≥60 minutes *vs.* <60 minutes/day; LPA all other variables are categorical.

## 4. Discussion

The purpose of this study was to examine the influence of urban design, neighbourhood built and social environment, and household and individual factors on the interdependent patterns of objectively measured MVPA, LPA and SB in children aged 10–14 years in the Canadian prairie city of Saskatoon. The first step in approaching this study’s goal was to descriptively depict the distribution of all intensities of activity (*i.e*., MVPA, LPA and SB) among children living in the three different types of neighbourhoods in Saskatoon (Table 2). It was clear that irrespective of the amount of MVPA and LPA accumulation, all children were sedentary for most of the day. This finding was reiterated when children’s MVPA and SB was categorized into weekday and weekend day accumulation, where boys and girls of all age groups were sedentary for most part of the day on both weekdays and weekends.

This accumulation of high SB is consistent with existing evidence [30], however, the descriptive results point towards another pattern as well. The Sedentary Behaviour Research Network recommends researchers to use the term ‘inactive’ to describe individuals who are not meeting age-specific PA guidelines [46]. In the distribution of activity intensities by different neighbourhoods it was observed that across all neighbourhoods even though on average children accumulated more than recommended MVPA of 60 minutes/day, they were consistently sedentary for most part of the day. Thus, it could be interpreted that children were ‘active and sedentary’ on the same day.

The weekday/weekend day segregation of MVPA and SB showed that in all age groups of boys and girls, the high accumulation of MVPA was limited to weekdays. However, boys and girls in all age groups were sedentary for the most part of the day on both weekdays and weekend days, a finding that reiterates that irrespective of children being active, they could still be sedentary. The descriptive findings not only establish the stable pattern of children’s sedentary lifestyle*,* but they also corroborate existing evidence that children are more active on weekdays [25,26,27]. 

The multilevel models explored the interrelationship between the three intensities of activity within the context of multilevel environmental exposures that influence these activities. Children who accumulated higher LPA were more likely to accumulate higher MVPA and less likely to accumulate higher SB. With experts in active living research currently developing integrated 24 hour movement behaviour guidelines for children and youth, the finding that LPA was associated in determining both MVPA and SB accumulation adds evidence to the concept of ‘whole day’ active living [32]. 

Although low weekend activity is well documented [25,26,27], the impact of this pattern and the factors that determine this pattern have not been explored in depth. To address this gap, weekend MVPA and SB were segregated and included in the multilevel models as individual variables. Children who accumulated higher MPVA during weekend days were more likely to accumulate higher MVPA and less likely to accumulate higher SB. Similarly, children who accumulated higher SB during the weekend days were more likely to accumulate higher SB*.* These findings portray the significance of weekend activity in the overall accumulation of MVPA and SB, and emphasize the need to develop weekend-specific active living interventions. 

Moreover, in determining the factors that drive low weekend activity, it is important to focus on the differential exposure to household/parental and peer environment between weekdays and weekend days. We observed that children who received frequent (>3 time/week) transportation from their families to a place with PA access, and children who frequently (>3 times/week) walked or biked with their peers (Section 2.5) were more likely accumulate higher MVPA. While parental and peer support was not categorized between weekdays and weekend days, it could be speculated that children who receive greater parental and peer support during the weekend days could very well be more active. Furthermore, parental and peer support are driven by more complex mechanisms. 

Parental support in providing transportation to a place with PA access could be related to vehicle ownership, which in turn is determined by family socioeconomic status. In terms of active transportation with peers, perceptions of safety come into play if children have to walk or bike together. With higher socioeconomic status neighbourhoods (suburban car-oriented curvilinear neighbourhoods) in Saskatoon having greater perceived safety [38], it could be interpreted that parental and peer support are connected to neighbourhood socioeconomic status and safety. These extrapolations underline the intricate multilevel relationships between social, built and economic factors in influencing activity accumulation. 

Finally, children living in fractured grid-pattern neighbourhoods (Figure 1) were less likely to accumulate higher MVPA than children living in grid-pattern neighbourhoods. The grid-pattern neighbourhoods surrounding the city centre, by virtue of their mixed land-use urban design (combination of commercial, residential, institutional establishments), possess greater density and diversity of destinations, are less car-oriented, and more pedestrian friendly in comparison with fractured-grid pattern neighbourhoods [35,38]. The observation that children living in mixed land-use neighbourhoods have a higher likelihood of accumulating higher MVPA corroborates current evidence that mixed land-use urban design is a strong predictor of PA among children and adolescents [8]. 

Nevertheless, even after conducting multivariable analyses that factored in multiple individual built environment features (e.g., safety from traffic and crime, density and diversity of destinations), only urban design emerged as a significant factor in influencing MVPA. This finding could be attributed to the close correlation between individual built environment features and urban design, where urban design (grid, fractured grid and curvilinear) not only encompasses, but also statistically supersedes all individual built environment features to holistically capture neighbourhood built environment. For example, although safety, density of destinations, attractiveness and universal accessibility are distinct facets of the built environment that could influence activity accumulation, our findings show that urban design which contains all these features plays a stronger role in influencing activity accumulation.

## 5. Strengths and Limitations

The primary strength of the study is the generation of evidence of the interdependence between objectively measured MVPA, LPA and SB. A challenge to this objectivity could be participants’ compliance for wearing accelerometers consistently. In our study sample, with the stringent criteria of at least four valid days, including at least one valid weekend day, the compliance rate was 72.74%. Apart from accelerometers, compliance could also be an issue for completing the questionnaires by participants, however, with trained project staff deploying the questionnaires in person, the compliance rate for completing the questionnaires was 100%.

Nevertheless, although objective intensities of activity were captured using accelerometers, there was a lack of social and spatial context related to activity accumulation. Hence, while associations between MVPA and SB accumulation and urban design, built and home environment have been established, these findings do not show *how* activity was accumulated within different environmental contexts or *where* (neighbourhood, indoor/outdoor, playground, recreational facility, *etc.*) activity was accumulated. 

Obtaining social and spatial context would enable the understanding of independent mobility of children, an active living indicator that is currently poorly understood [47]. Studies are now emerging which utilize ecological momentary assessments and global positioning systems to understand the complex social and spatial associations of activity accumulation [48,49]. These advances, when combined with accelerometry, would provide the methodological depth to tease out the complex pathways that determine activity accumulation. Finally, as this study is based on cross-sectional data, causal inferences cannot be delineated from the findings.

## 6. Conclusions

This study highlights the complexity of active living research in capturing the interplay between objectively derived activity intensities (*i.e*., MVPA, LPA and SB) and how these activities interact with each other within the wider context of multilevel environmental exposures. The study depicted the interdependent relationships between MVPA, LPA and SB, with the findings pointing towards the development of active living interventions that conceptualize these activity intensities together. The study also emphasizes the need to understand weekend day activity accumulation to inform active living strategies. In terms of the environment, the findings showed that a multilevel set of factors, including urban design, and parental and peer support need to be considered in developing active living interventions.

## Supplementary Files

Supplementary File 1
