# Supplementary material for: Capturing the Interrelationship between Objectively Measured Physical Activity and Sedentary Behaviour in Children in the Context of Diverse Environmental Exposures
Source: Int J Environ Res Public Health. 2015 Sep 7;12(9):10995–1011. doi: 10.3390/ijerph120910995 (PMC4586657; doi:10.3390/ijerph120910995)
Supplement: Supplementary File 1 [file ijerph-12-10995-s001.zip › SCHK Questionnaire.pdf]

University of Saskatchewan - Smart Cities, Healthy Kids Research Project

University of Saskatchewan

NOTE TO ALL STUDENTS:

This is a survey with questions about youth physical activity and nutrition. Your answers will help the Smart Cities, Healthy Kids Project learn about how kids like to stay active and what may prevent them from participating in activities. The survey will take approximately 30 minutes to complete.

NBHD-SCHL	CHILD ID
0	0
1	1
2	2
3	3
4	4
5	5
6	6
7	7
8	8
9	9

MARKING INSTRUCTIONS

- **Use an HB pencil only.**
- **Do not use ink, ball point, or felt tip pens.**
- **Make solid marks that fill the response completely.**
- **Erase cleanly any marks you wish to change.**

CORRECT: ● **INCORRECT:** ⊘ ⊠ ◒ ◔

01091

DO NOT WRITE IN THIS BOX

Section A : About Me and My Family

1. I am a .... ○ Boy ○ Girl
2. What grade are you in? .... ○ 5 ○ 6 ○ 7 ○ 8
3. How old are you? .... ○ 9 ○ 10 ○ 11 ○ 12 ○ 13 ○ 14 ○ 15
4. What is the name of your School? .... ____________
5. Please write your **Street Address** .... ____________ (eg. 123 Main Street)

   Please write your **Postal Code** .... ____________ (eg. S7L1E6)

This information will only be used to confirm your neighborhood.

6. Do you identify as an Aboriginal person (First Nations, Métis, Inuit)? ○ Yes ○ No
7. Where do you live most of the time?
   - ○ Both parents (biological or adopted)
   - ○ Mother only
   - ○ Father only
   - ○ Mother part time/ Father part time
   - ○ Other relative (Grandmother, Aunt, Uncle, etc.)
   - ○ Group home or Foster home
   - ○ Other
8. How many brother and sisters do you have that live with you right now?
   - ○ None
   - ○ 2
   - ○ 4
   - ○ 6
   - ○ 1
   - ○ 3
   - ○ 5
   - ○ 7 or more
9. What is the highest level of schooling that your father completed?
   - ○ Less than high school
   - ○ Finished high school
   - ○ Trade School (ex., mechanic, technician, journeyman, librarian)
   - ○ Some university
   - ○ Finished university
   - ○ Don't know or Doesn't Apply
   - ○ Other

10. What is the highest level of schooling that your mother completed?
- ◯ Less than high school
- ◯ Finished high school
- ◯ Trade School (ex. mechanic, technician, journeyman, librarian)
- ◯ Some university
- ◯ Finished university
- ◯ Don't know or Doesn't Apply
- ◯ Other

11. Would you describe your family's money situation as (please choose only one answer):
- ◯ Wealthy
- ◯ Average
- ◯ Difficult
- ◯ Poor
- ◯ Don't know

	Excellent	Very Good	Good	Fair	Poor
12. In general would you say that your health is:	◯	◯	◯	◯	◯

13. Is it hard for you to do physical activities because of health problems that have lasted 6 months or longer?
- ◯ Yes
- ◯ No
- ◯ Don't Know/Not Sure

14. This next question is about your weight. Choose the answer that is closest to how you feel:
- ◯ I think I'm overweight (by 5 pounds or more)
- ◯ I think my weight is Okay
- ◯ I think I'm underweight (by 5 pounds or more)

15. How well are you doing in school this year?
- ◯ Above average
- ◯ Average
- ◯ Below average

16. This year, where have most of your marks been?
- ◯ 90% or higher
- ◯ 80 - 89%
- ◯ 70 - 79%
- ◯ 60 - 69%
- ◯ 50 - 59%
- ◯ 49% or lower

Section B. About My Family

17. In the **last 30 days** how often did you see any of your family members participate in sports or exercise?
- ◯ Never
- ◯ Once or Twice
- ◯ 3 or 4 times
- ◯ 5 times or more

18. In the ~~last 30 days~~ how often have your family members (mom, dad, grandma, grandpa, brothers, sisters etc.)...

	Never	Once or Twice	3 or 4 Times	5 times or more
a. Said that you were good at the physical activities that you did?	◯	◯	◯	◯
b. Got upset about the physical activities that you did?	◯	◯	◯	◯
c. Said that physical activity was good for you?	◯	◯	◯	◯
d. Offered to be active with you? (ex. ride bikes, throw a ball, etc.)	◯	◯	◯	◯
e. Ordered you to be active?	◯	◯	◯	◯
f. Paid for programs or equipment to help you stay active?	◯	◯	◯	◯
g. Helped you to learn or improve the skills you use when you are being active (ex. for playing sports)?	◯	◯	◯	◯
h. Talked to you about how much fun physical activity is?	◯	◯	◯	◯
i. Watched you participate in physical activity or play sports?	◯	◯	◯	◯
j. Encouraged you to do sports or physical activity?	◯	◯	◯	◯
k. Provided transportation to a place where you can do physical activity?	◯	◯	◯	◯
l. Done a physical activity or played sports with you (ex. walked or biked)?	◯	◯	◯	◯

19. During a typical week, how often:	Never	1-2 days	3-4 days	5-6 days	Every Day	I have no siblings
a. Do your brother(s) and/or sister(s) encourage you to do sports or exercise?	◯	◯	◯	◯	◯	◯
b. Do your brother(s) and/or sister(s) do physical activity or play sports with you?	◯	◯	◯	◯	◯	◯

Section C. About My Friends

20. During a typical week, how often:	Never	1-2 days	3-4 days	5-6 days	Every Day
a. Do your friends encourage you to do sports or physical activities?	◯	◯	◯	◯	◯
b. Do your friends do physical activities or play sports with you?	◯	◯	◯	◯	◯
c. Do your friends or classmates tease you about not being good at physical activities or sports?	◯	◯	◯	◯	◯
d. Do your friends ask you to walk or to bike to school or to a friend's house?	◯	◯	◯	◯	◯
e. Do your friends tell you that you are doing well in physical activities or sports?	◯	◯	◯	◯	◯

21. In the **last 30 days**, how often:	Never	1-2 days	3-4 days	5-6 days	Every Day
a. Did you see any of your friends participate in physical activity?	◯	◯	◯	◯	◯
b. Did any of your friends say that physical activity was good for you?	◯	◯	◯	◯	◯
c. Did any of your friends force you to be active?	◯	◯	◯	◯	◯
d. Did any of your friends help you to learn/improve the skills that you use to be active?	◯	◯	◯	◯	◯
e. Did any of your friends come to watch you when you were doing physical activity?	◯	◯	◯	◯	◯
f. Did any of your friends say that you were good at doing physical activity?	◯	◯	◯	◯	◯

22. How many of your closest friends exercise regularly?

◯ None ◯ 1-2 Friends ◯ 3-4 Friends ◯ 5 or More Friends

Section D: Reasons I might not be active

23. How often have the following things kept you from being active?

	Never	Rarely	Sometimes	Often	Always
a. I felt lazy	◯	◯	◯	◯	◯
b. I didn't feel like it	◯	◯	◯	◯	◯
c. It makes me look uncool	◯	◯	◯	◯	◯
d. I didn't have the money	◯	◯	◯	◯	◯
e. The activity is not close to my home	◯	◯	◯	◯	◯
f. I couldn't get a ride	◯	◯	◯	◯	◯
g. I didn't have enough time	◯	◯	◯	◯	◯
h. I had too much homework	◯	◯	◯	◯	◯
i. It gets in the way when I want to hang out with friends	◯	◯	◯	◯	◯
j. I was injured	◯	◯	◯	◯	◯
k. The weather was bad	◯	◯	◯	◯	◯
l. The equipment was broken	◯	◯	◯	◯	◯
m. I had family responsibilities (ex. chores, looking after brother or sister, etc.)	◯	◯	◯	◯	◯
n. The area was not safe	◯	◯	◯	◯	◯
o. It was too crowded	◯	◯	◯	◯	◯

Section E: About Me and Activities

24. In an average week, when you are in school, on how many days do you go to gym classes?

◯ 1 ◯ 2 ◯ 3 ◯ 4 ◯ 5

25. In an average week, about how many hours a day, in total, do you watch TV, videos/DVDs, play video games or use the computer?

◯ I don't have any screen time in a day ◯ Less than 1 hour a day ◯ 1 to 2 hours a day
◯ 3 to 4 hours a day ◯ 5 to 6 hours a day ◯ 7 or more hours a day

26. In the **last 30 days** how often have you:	Never	1 or 2 times	3 or 4 times	5 times or more
a. Played sports or done physical activities **without** a coach or an instructor (ex. biking, skateboarding, etc.)?	◯	◯	◯	◯
b. Played sports **with** a coach or instructor other than in gym class? (ex. swimming lessons, baseball, hockey, etc.)?	◯	◯	◯	◯
c. Taken part in dance, gymnastics, karate, or other groups or lessons other than in gym class?	◯	◯	◯	◯
d. Taken part in art, drama, or music groups, clubs, or lessons?	◯	◯	◯	◯
e. Taken part in clubs or groups such as Guides or Scouts, 4-H club, community, church or other religious groups?	◯	◯	◯	◯
f. Done a hobby or craft (ex. drawing, model building, etc.)?	◯	◯	◯	◯

01091

Section F: Where I go to do activities

27. Here is a list of places where people can exercise. Which ones do you go to that are somewhere you could **walk** or **ride your bike** to easily? (Mark all that apply)

- ○ Fitness facility
- ○ Basketball court
- ○ Bike lane or trails
- ○ Golf Course
- ○ Public park
- ○ Running track
- ○ Skating rink/outdoor rink
- ○ Swimming Pool
- ○ Walking/hiking trails
- ○ Tennis courts
- ○ Dance studio
- ○ Playing field (soccer, football, softball, etc.)
- ○ Other
- ○ There are no places close to my house

28. For the places that you marked in **Question 27**, how often would you walk or bike to these places in a typical month?

○ Once a month ○ 2-4 times a month ○ 5 or more times a month

29. How long does it take you to walk to the closest park from your house?

○ Less than 5 mins ○ 5-10 mins ○ 10-20 mins ○ More than 20 mins

30. In the **last 30 days**, how often have you walked or biked to school?

- ○ Never
- ○ Once a week
- ○ Two to four times a week
- ○ Every day

31. When you didn't walk or bike to school was it because (Mark as many reasons as apply) ...

- ○ School is too far from my house
- ○ I didn't have time
- ○ My parents or guardians drove me
- ○ The weather wasn't good for walking or biking
- ○ I take the bus instead
- ○ I was sick or injured and couldn't walk or bike
- ○ It was not safe
- ○ Other - explain ____________________

32. In a typical week, how much time do you spend walking	**Less than an hour**	**About an hour**	**1-5 hours**	**More than 5 hours**
a. For fun (ex. just walking with friends)	○	○	○	○
b. To do errands(ex. going to get the mail or to the store)	○	○	○	○
c. To get someplace (ex. to the park or playground)	○	○	○	○

33. How often do you walk or bike to each of the following places in a typical week?

	**Never**	**1-2 times**	**3-4 times**	**5 or more times**
a. Corner store/Convenience Store	○	○	○	○
b. Bus stop	○	○	○	○
c. Library	○	○	○	○
d. Church or place of worship	○	○	○	○
e. Friend or relatives' house	○	○	○	○
f. Restaurant	○	○	○	○
g. Shopping Mall	○	○	○	○
h. Grocery store	○	○	○	○
i. Entertainment outlet (ex.Video store, Ruckers, Movie Theatre, etc.)	○	○	○	○

34. If you answered **'Never'** to any of the options in **Question 33,** mark as many of the following reasons that apply....

- ◯ It is too far from my house
- ◯ I didn't have time
- ◯ My parents or guardians drove me
- ◯ The weather wasn't good for walking or biking
- ◯ I took the bus instead
- ◯ I was sick or injured and couldn't walk or bike
- ◯ It was not safe
- ◯ Other - explain ______________________________

35. Please fill in the circle beside each item that you have in your home, yard or apartment complex. (Mark all that apply).

- ◯ Treadmill, Stair climber, Stationary Bike
- ◯ Bicycle (for outside)
- ◯ Trampoline
- ◯ Running shoes
- ◯ Swimming Pool
- ◯ Step aerobics, Slide aerobics
- ◯ Ice Skates or Roller Blades
- ◯ Balls or Racquets
- ◯ Weight lifting equipment (ex. free weights, Nautilus, Universal)
- ◯ Snowboard
- ◯ Canoe, row boat, kayak
- ◯ Skis (snow or water)
- ◯ Basketball hoop
- ◯ Backyard rink
- ◯ Hockey nets or sticks
- ◯ Wii fit or Dance Dance revolution
- ◯ I have no equipment

36. How much do you agree with the following statements?

	**Strongly Disagree**	**Somewhat Disagree**	**Somewhat Agree**	**Strongly Agree**
a. At home there are enough supplies and pieces of sports equipment (like balls, bicycles, skates) to use for physical activity....................	◯	◯	◯	◯
b. It is difficult to walk or jog in my neighbourhood because of things like traffic, no sidewalks, gangs, etc.	◯	◯	◯	◯
c. There are playgrounds, parks, or gyms, that are close to my home or that I can get to easily	◯	◯	◯	◯
d. It is safe to walk or jog in my neighbourhood during the day	◯	◯	◯	◯
e. It is safe to ride my bike on the road in my neighbourhood	◯	◯	◯	◯

37. Please fill in the circle beside each item on the list that is found in your neighbourhood. (Mark all that apply).

- ◯ Sidewalks
- ◯ Heavy Traffic
- ◯ Hills
- ◯ Street lights
- ◯ Dogs that are loose
- ◯ Enjoyable scenery/Parks
- ◯ Frequently see people walking or exercising
- ◯ High Crime

38. How **safe** do you feel **walking** in your neighbourhood during the day?

◯ Very Unsafe ◯ Pretty Unsafe ◯ Safe ◯ Pretty Safe ◯ Very Safe

39. How **safe** do you feel **riding your bike** in your neighbourhood during the day?

◯ Very Unsafe ◯ Pretty Unsafe ◯ Safe ◯ Pretty Safe ◯ Very Safe

40. How **safe** do you feel **crossing the street** in your neighbourhood during the day?

◯ Very Unsafe ◯ Pretty Unsafe ◯ Safe ◯ Pretty Safe ◯ Very Safe

41. How **easy** is it to **cross the street** in your neighbourhood during the day?
   - Very hard
   - Pretty hard
   - Pretty easy
   - Very Easy

Section G: Food I eat

42. In the **last 30 days**, how often did you eat breakfast before you came to school?
   - Seldom or never
   - 1-2 times a week
   - 3-4 times a week
   - 5 times a week

43. In the **last 30 days**, how often did you go hungry because there was not enough food?
   - Never
   - Rarely
   - Sometimes
   - Most of the time
   - Always

44. In the **last 30 days**, how many times per day did you eat fruit?
   - I did not eat fruit in the last 30 days
   - Less than one time per day
   - 1 time per day
   - 2 times per day
   - 3 times per day
   - 4 times per day
   - 5 or more times per day

45. In the **last 30 days**, how many times per day did you eat vegetables?
   - I did not eat vegetables in the last 30 days
   - Less than one time per day
   - 1 time per day
   - 2 times per day
   - 3 times per day
   - 4 times per day
   - 5 or more times per day

46. In the **last 30 days**, how many times per day did you drink pop, fruit drinks or energy drinks?
   - I did not drink pop, fruit drinks or energy drinks in the last 30 days
   - Less than one time per day
   - 1 time per day
   - 2 times per day
   - 3 times per day
   - 4 times per day
   - 5 or more times per day

47. In the **last 30 days**, how many times per day did you drink milk (including soy milk and rice milk)?
   - I did not drink milk in the last 30 days
   - Less than one time per day
   - 1 time per day
   - 2 times per day
   - 3 times per day
   - 4 times per day
   - 5 or more times per day

48. In the **last 30 days**, how many times per day did you eat food from a fast food restaurant; include food that you ordered in as take out (ex. McDonalds, KFC, Taco Time, Pizza Hut, etc.)?
   - I did not eat fast food in the last 30 days
   - Less than one time per day
   - 1 time per day
   - 2 times per day
   - 3 times per day
   - 4 times per day
   - 5 or more times per day

49. What do you think your school can do to help kids your age become more physically active?
____
____
____
____
____

50. Can you tell us what you think would make the park closest to your house better?
____
____
____
____
____

51. Is there anything else you would like to share with us about being physically active?
____
____
____
____
____

THE END

☺ THANK YOU FOR YOUR HELP! ☺
