# Supplementary material for: Capturing the Interrelationship between Objectively Measured Physical Activity and Sedentary Behaviour in Children in the Context of Diverse Environmental Exposures
Source: Int J Environ Res Public Health. 2015 Sep 7;12(9):10995–1011. doi: 10.3390/ijerph120910995 (PMC4586657; doi:10.3390/ijerph120910995)
Supplement: Supplementary File 1 [file ijerph-12-10995-s001.zip › ijerph-93115-suppl.pdf]

*Supplementary Information*

# Capturing the Interrelationship between Objectively Measured Physical Activity and Sedentary Behaviour in Children in the Context of Diverse Environmental Exposures

**Table S1**. Analyses to determine daily SB cut-point for multilevel logistic regression.

**Table S1a.** Sensitivity analysis testing daily SB cut-points protective of overweight and/or obese status.

| SB Cut-Points | OR | CI |
|---|---|---|
| SB minutes/day (<360 *vs*. ≥360) | 0.000 | 0.000–0.000 |
| SB minutes/day (<420 *vs*. ≥420) | 0.213 | 0.270–1.667 |
| SB minutes/day (<480 *vs*. ≥480) * | 0.428 | 0.188–0.975 |
| SB minutes/day (<540 *vs*. ≥540) * | 0.515 | 0.301–0.882 |
| SB minutes/day (<600 *vs*. ≥600) | 0.542 | 0.292–1.006 |
| SB minutes/day (<660 *vs*. ≥660) | 0.503 | 0.117–2.157 |

**Table S1b.** Multivariable logistic regression depicting the selected SB cut-point's association with overweight and/or obese status.

| Variables | OR | CI |
|---|---|---|
| SB minutes/day (<480 *vs*. ≥480) * | 0.424 | 0.182–0.989 |
| Boys *vs*. Girls | 1.092 | 0.658–1.815 |
| Age 11 *vs*. Age 10 | 0.877 | 0.430–1.789 |
| Age 12 *vs*. Age 10 | 1.217 | 0.576–2.570 |
| Age 13 *vs*. Age 10 | 1.571 | 0.691–3.571 |
| Age 14 *vs*. Age 10 | 0.711 | 0.246–2.050 |
| Mother's Education (University degree *vs*. all other) ** | 0.521 | 0.311–0.870 |

SB: sedentary behaviour; OR: odds ratio; CI: confidence interval; * $p < 0.05$; ** $p < 0.01$; wear-time was controlled for in both sensitivity analyses and the multivariable logistic regression analysis.

© 2015 by the authors; licensee MDPI, Basel, Switzerland. This article is an open access article distributed under the terms and conditions of the Creative Commons Attribution license (http://creativecommons.org/licenses/by/4.0/).
